# Patient treatment pathways of multidrug-resistant tuberculosis cases in coastal South India: Road to a drug resistant tuberculosis center

**DOI:** 10.12688/f1000research.17743.3

**Published:** 2020-05-27

**Authors:** Priya Rathi, Kalpita Shringarpure, Bhaskaran Unnikrishnan, Abhinav Pandey, Abhirami Nair

**Affiliations:** 1 Department of Community Medicine, Kasturba Medical College, Mangalore, Manipal Academy of Higher Education, Manipal, Karnataka, India; 2 Department of Preventive and Social Medicine, Medical College Baroda, Baroda, Gujarat, India; 3 Kasturba Medical College, Mangalore, Manipal Academy of Higher Education, Manipal, Karnataka, India

**Keywords:** Diagnostic delay, Treatment delay, Patient delay, Health care system delay Programmatic management of Drug resistant TB, detection rate, Private health care facility, Public health care facility.

## Abstract

**Background**: Delays in initiating multidrug-resistant tuberculosis (MDR TB) treatment adds risk to individual patients and the community due to disease progression, and on-going transmission. The Government of India offers free TB diagnosis and treatment, however many presumptive MDR TB patients wander within the Indian healthcare system and delay accessing the programme. To improve access to care, it is imperative to understand the treatment pathways taken by MDR TB patients. We aimed to describe the diagnostic and treatment pathway taken by presumptive MDR TB patients registered under Programmatic Management of Drug-resistant TB Program.

**Methods**: We conducted a cross-sectional study amongst patients registered during August 2016 – April 2017 at one District Drug Resistance Tuberculosis centre of Dakshina Kannada district in Karnataka, India. A semi-structured questionnaire was used to collect the number, type (private and public sector), and dates of healthcare facilities (HCFs) visits prior to the initiation of MDR TB treatment. Delays in pathway were measured in days and summarised as median and interquartile range (IQR), from the date of onset of illness until the initiation of MDR TB treatment.

**Results**: We found that patients preferred private HCFs; however, due to lack of treatment and unaffordability they shifted to public HCFs. Median delay to register under the program was more in private HCFs (180 days) in comparison with public HCFs (120 days). We also found that the detection rates were much higher in public HCFs (80%).

**Conclusion**: The present study found that there was substantial patient delay and total delay in diagnosis and treatment of MDR TB patients. Private HCF was first point of contact for most of the patients; however the diagnostic rate was high in public HCF. The government should involve private HCFs to provide standard diagnostics and treatment to the patients seeking a private facility.

## Introduction

Multidrug-resistant tuberculosis (MDR TB), is defined as tuberculosis (TB) bacilli resistant to at least two first-line drugs — rifampicin and isoniazid. According to the Global TB Report 2019 published by the World Health Organization (WHO), India ranks first globally in the burden of TB and MDR TB with an incidence rate of 27% and 24% of global burden respectively ^1, 2^. Nearly 3% of new TB cases and 12% of previously treated patients in India are MDR TB ^2, 3^.

To reduce transmission of MDR TB, the Government of India developed and implemented a national policy for the programmatic management of drug-resistant TB (PMDT) in 2007 under the Revised National Tuberculosis Control Programme (RNTCP), now renamed National Tuberculosis Elimination Programme(NTEP) ^4^. The strategies and objectives of PMDT complement the National Strategic Plan for TB Elimination (2017–2025), and include treatment of MDR TB, enhancing laboratory systems for faster diagnosis, and offering social protection and supportive systems to ensure uninterrupted treatment with shorter, less toxic regimens ^4– 6^.

Delays in MDR TB diagnosis and appropriate treatment initiation not only impacts individual patients, through advance disease progression, additional costs, and poor quality of life; but also in the community through increased risk of ongoing transmission to other individuals ^7, 8^. The time taken to seek care, type of health care facility being sought and reasons for shifting from one health care facility to other are important factors for understanding delays in diagnosis, treatment initiation, and poor outcomes. Describing health seeking behaviours and pathways taken by persons presumed to be MDR TB case is a vital step in developing and implementing interventions that bridge the gap between timely diagnosis and treatment, and policies that improve the overall health system.

In India, approximately 20% of the patients in need of MDR TB treatment actually receive it, and among those who do receive and start treatment, less than half (48%) complete it successfully ^2, 3^. These poor outcomes are largely due to lost to follow-up and premature deaths. In 2017, the proportion of deaths during MDR TB treatment in India was higher than the global average (21% vs. 14%) ^3^.

Therefore, in this study we aimed to determine the health-seeking pathway of presumptive MDR TB patients prior to treatment at the PMDT centre. We also assessed the median time taken, and reasons for shifting from one heath care facility (HCF) to another.

## Methods

### Study setting

The Dakshina Kannada(DK) district in Karnataka provinces of India includes 79 primary health centers (PHCs), five secondary referral hospitals (Taluk hospitals), one tertiary referral hospital (Wenlock Government hospital) and over 500 private practitioners. Mangalore is a second major city and is headquarters of DK, with a population of six lakhs. This city is one of the major center for healthcare, with a inflow of patients not only from Mangalore but also from adjacent states of Kerala and Tamil Nadu.

We conducted a cross-sectional study of all patients registered during August 2016 – April 2017 at one District Drug Resistance Tuberculosis Centre (DDR-TBC) of DK district which also caters to two neighbouring districts of Chikmagalur and Udupi in Karnataka, India. It has ten inpatient beds, facilities for diagnosis, pre-treatment evaluation and treatment of DRTB, monitoring complications associated with second-line anti-tuberculosis treatment. After inpatient care to initiate treatment (approximately two weeks), the patient is released for continuation of care at Peripheral Health Institute (PHI) on an outpatient basis, with the help of identified treatment supporter throughout the community.

After obtaining Institutional Ethics Committee (IEC) approval from Kasturba Medical College Mangalore (IEC KMC MLR 11-16/328) and permission from DDR-TBC, all patients registered under DDR-TBC were asked to enrol in the study. A line list was taken from DDR-TBC on daily basis, and the patients present in DDR-TBC were approached by the Principal Investigator (PI), The purpose of the study was explained to the patients in their vernacular language and written informed consent was obtained from patients. Those who were illiterate, informed consent process was conducted in front of literate impartial witness ^9^. PI collected data using a questionnaire to interview the patients.

### Data collection

Face to face interviews were guided by a semi-structured questionnaire, which had been developed based on a literature review and the content was validated by experts in epidemiology (CDC Atlanta), a PMDT medical officer, and a layperson (local ground staff member working in the tertiary care hospital). Following this, the tool was field tested in two patients for ease of administration, who were not added in analysis, The interview enquired about the various healthcare facilities (HCFs) visited by the patient from the time they experienced TB symptoms, designated as a presumptive MDR patient (designated as a presumptive MDR patient which by definition refers to the following patients: TB patients found positive on any follow-up sputum smear examination during treatment with first line drugs including treatment failures; - TB patients who are contacts of DR TB; - previously treated TB patients; - new TB patients with HIV co-infection ^10^), until they registered for treatment at the PMDT centre. We noted the time interval and reason for shifting from one facility to another. All information was penned down on the questionnaire at the time of the interview without any audio or video recording. We also validated the starting point with the reports and patient file from the DDR-TBC to look for HIV coinfection, previous lab reports, history of contact, previous treatment history, if available.

### Data analysis

Data collected was entered and analyzed using Statistical Package for Social Science (SPSS) version 11.5. Kolmogorov-Smirnov test was done to find the normality of data. Results were expressed in median and inter-quartile range (IQR). Chi-square test was performed to find out the association between type of HCF visited by patients and reasons for shifting from one HCF to the other. The patient treatment pathway has been used to express health seeking behavior of the patients and was created using Adobe Illustrator trial version. (Adobe Creative Cloud- Illustrator. Available from: http://adobe.ly/28QoDIL) The pathway was created using vector images with the visits being represented with different colors and different kinds of lines used for the diagnostic status of the patient. The meanings of both the lines as well the colors have been explained in the legends accompanying the pathways. Moreover, the number of patients shifting between HCF has been represented using the numbers accompanying the respective lines.

***Operational definitions.*** *Multidrug resistant TB (MDR-TB):* Patients with sputum-smear positive pulmonary TB, and at least one *M. tuberculosis* isolate with demonstrated resistance to at least isoniazid and rifampicin

*Presumptive MDR-TB patients:* A presumptive MDR patient which by definition refers to the following patients: TB patients found positive on any follow-up sputum smear examination during treatment with first line drugs including treatment failures; - TB patients who are contacts of DR TB; - previously treated TB patients; - new TB patients with HIV co-infection ^10^.

*Pathway:* The various type of HCF visited by a presumptive MDR patient before registering for PMDT treatment in a chronological sequence. The various HCF were merged into two broad types: private and public health care sectors. Public HCFs include Primary Health Centers (PHCs) and Public Referral Hospitals (PRHs), including secondary and tertiary referral centers. Private HCF include secondary and tertiary referral center participating in Revised National Tuberculosis Control Program, non-participating allopathic clinics and practitioners (registered and unregistered) and Ayurveda Yoga Unani Sidda Homeopathy (AYUSH) practitioners ^11^.

*Time delays in the health care pathways* ^12^: The total delay is the time interval from the onset of illness until the initiation of anti MDR-TB drugs. It is the sum of two-time intervals: 1) diagnostic delay (time interval between the onset of symptoms and labelling of the patient as a MDR-TB patient); 2) treatment delay (time interval between MDR-TB diagnosis and initiation of anti MDR-TB drugs).

The total delay is also the sum of patient delay (time interval between onset of symptoms and presentation to first health care provider) and healthcare system delay (time interval between the date of health-seeking behavior at a health care provider and the initiation of anti MDR-TB treatment), since it can be attributed to these types of delay.

*Reasons for shifting from one HCF to another:* These were the option provided to the patients-

1. Treatment not available, the appropriate treatment for the symptoms was unavailable in that HCF;
2. Treatment not affordable, cost of treatment was beyond the paying capacity of patient and their family;
3. Referred, patient was asked to visit another HCF for review/consultation or appropriate treatment;
4. Not satisfied, symptoms did not alleviate or the patient perceived that the services being provided were inadequate. Also, along with this, they were also asked of any other reasons.

## Results

During the study period, a total of 55 patients were initiated on treatment at the DDR-TBC; however only 40 patients consented to participate in the study. The mean age in our study was 40 years (SD: 13.9). There were 28(70%) male patients and 12 (30%) female patients. In total, 35(87.5%) patients were educated till primary level, while only 5(12.5%) of patients were illiterate. A total of 26 (65%) of the study participants belonged to rural areas while only 14(45%) patients lived in an urban area.

### Details of first visit and shift from one HCF to other HCF

**Figure 1.**
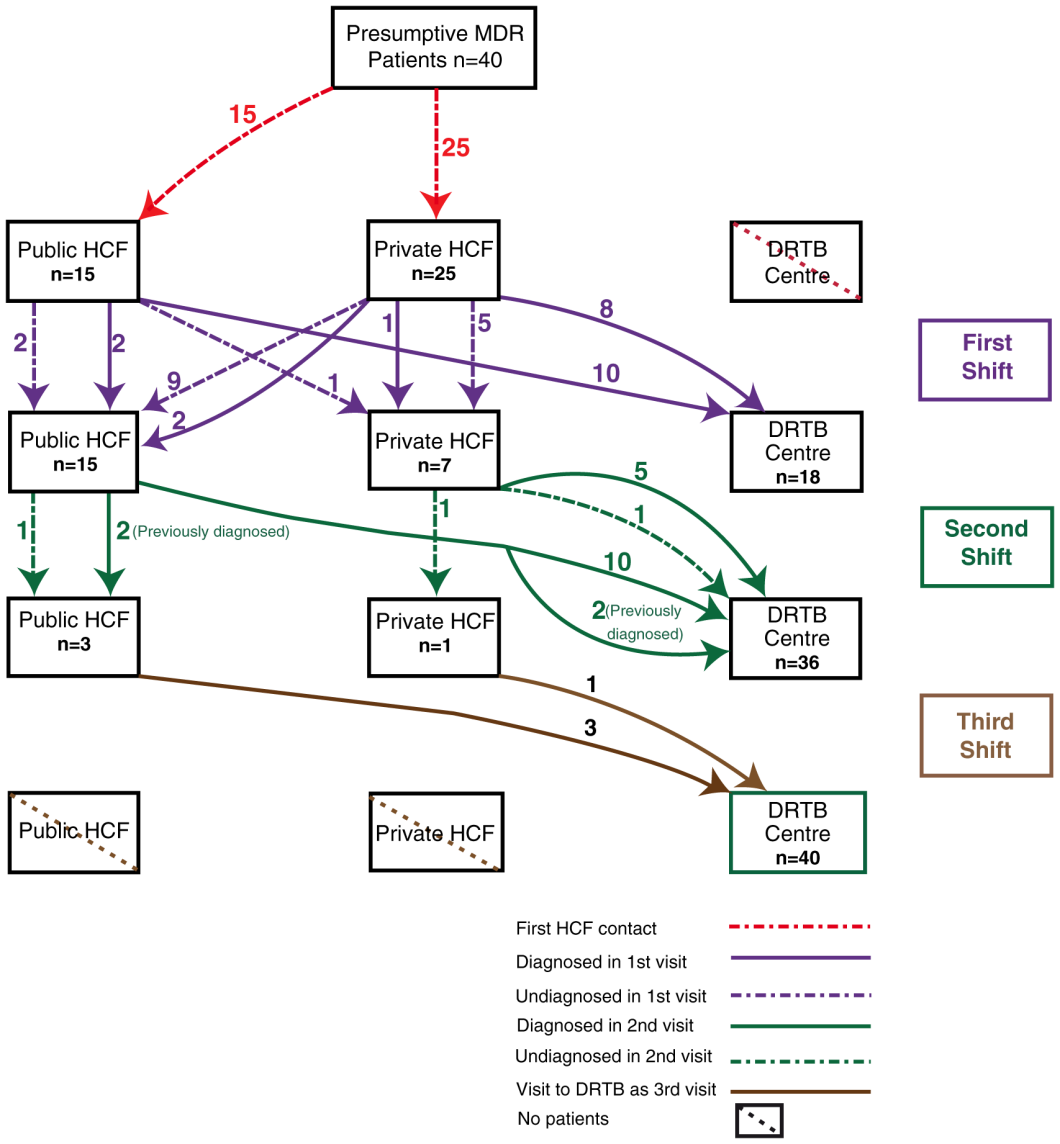
Patient diagnostic and treatment pathway.

**Figure 2.**
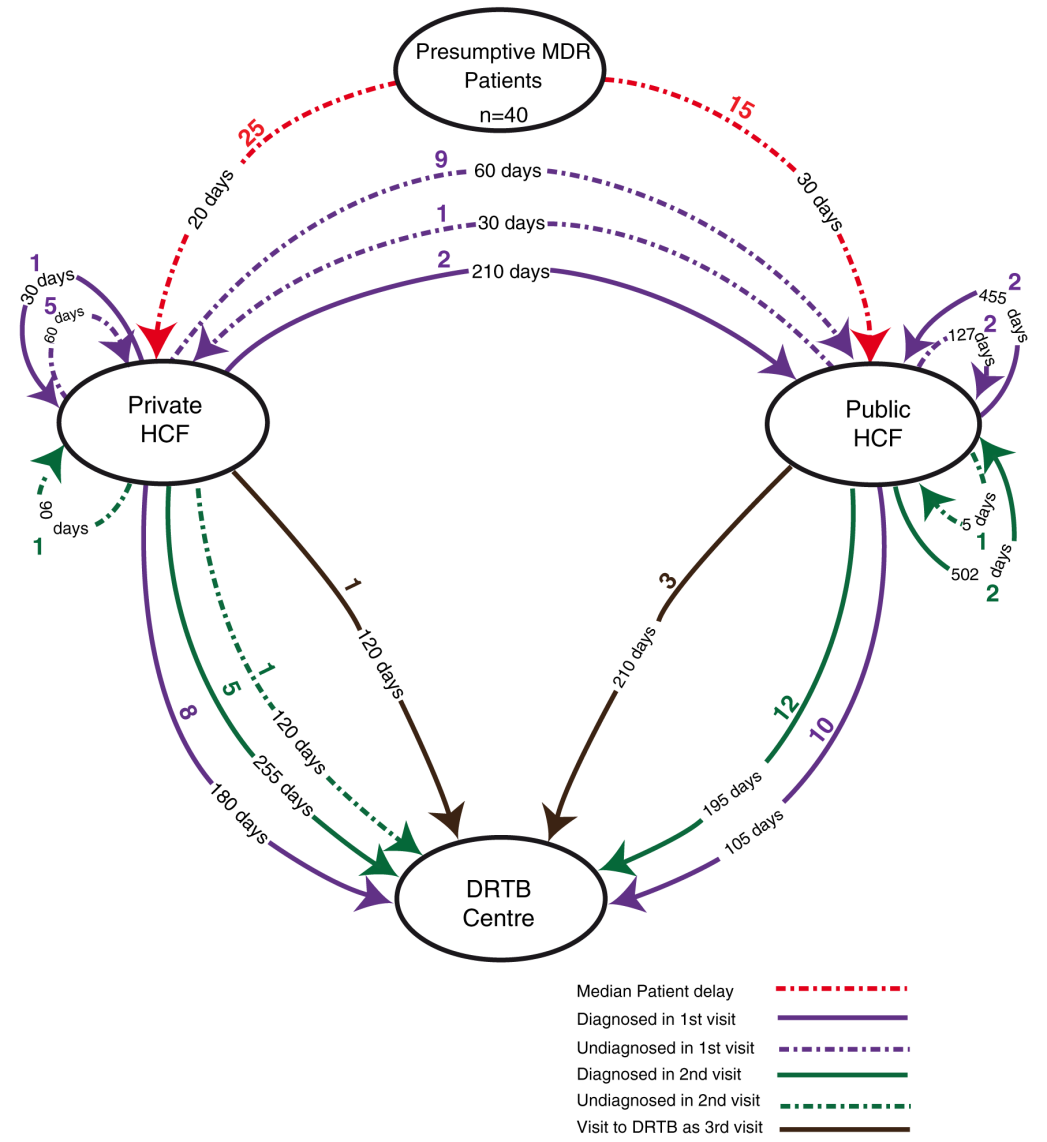
Median time delay in treatment pathway of MDR Patients.

Out of 40 patients interviewed, 15(37.5%) went to a public HCF and 25(62.5%) went to a private HCF as their first clinical encounter ( Figure 1 and Figure 2). In total, 23 (57.5%) were diagnosed with MDR TB during this encounter.

Amongst the 15 patients who went to the public HCF at the first encounter, 12 (80%) were diagnosed with MDR TB and were transferred directly to the DDR-TBC., whilst the remaining three patients required additional visits before MDR diagnosis (two went to public HCF and one went to private HCF).

Amongst the 25 patients who went to the private sector at the first clinical encounter, 11 (44%) were microbiologically diagnosed with MDR TB, and were referred directly to the DDR-TBC. The remaining 14(66%) required addition visits before MDR TB diagnosis (nine (36%) went to public HCF and five (20%) went to private HCF).

### Details of second visit and shift from one HCF to other HCF

All 40 patients underwent a second visit: 18(45%) went to DDR-TBC, 15(37.5%) opted for public HCF, while seven (17.5%) went to private HCF. Out of the 17 undiagnosed patients, 14 (82.3%) were diagnosed in second visit with MDR TB.

Out of the 15 who went to public HCF for second visit, four were already diagnosed (two from public HCF and two from private HCF) and 11(73.3%) were undiagnosed. Of the 11 undiagnosed, ten (91%) were diagnosed on this visit and were referred to the DDR-TBC. One undiagnosed patient went to another public sector. Out of four previously diagnosed patients, two were referred to DDR-TBC and two again shifted to another public sector HCF. Hence a total of 12(80%) patients out of 15 shifted to DDR-TBC and three visited other public HCF.

Seven patients went to private HCF for a second visit. Out of these seven, one was previously diagnosed in other private institution and six were undiagnosed (five came from private HCF and one from public HCF). In this visit, of the six patients who were undiagnosed, four were diagnosed. Five patients (one previously diagnosed and four newly diagnosed) visited DDR -TBC from a private HCF. Two still remained undiagnosed. One went to the DDR-TBC. The other undiagnosed patient visited another private sector HCF.

Overall, 18 patients (12(66.6%) from public HCF and six(33.3%) from private HCF) visited DDR-TBC in their third visit.

### Details of third and fourth visit and third shift from one HCF to other HCF

Of the 40 patients, 22 (55%) patients underwent a third visit to HCF. Among them, 18(82%) came to DDR-TBC, three went to public HCF while one went to the private sector. Out of the three who went to public HCF, one was undiagnosed and two were previously diagnosed (first visit). The undiagnosed case was diagnosed and referred to the DDR-TBC. The two previously diagnosed patients also went to DDR-TBC in their fourth visit.

One undiagnosed patient from the private HCF was diagnosed and referred to DDR-TBC in the fourth visit.

### Time delay in treatment pathway of MDR patients

The median (IQR) patient delay was found to be 25 (10, 60) days. For patients contacting public HCF as their first point of health care contact, the median patient delay was 30 days, while for those contacting private HCF, it was found to be 20 days.

In the first visit among the 23 patients who were diagnosed to have MDR TB, the median delay in reaching the DDR-TBC after contacting the first HCF was 120 (30, 240) days. Median (IQR) delay in a public health care facility was 105 (60, 382) days with the highest being 1825 days, while in a private health care facility the median delay was 180 (10, 240) days with the highest being 300 days.

Among the patients diagnosed in second visit, the median delay in reaching DDR-TBC was 210 (82,270) days from the day of first HCF contact. In public HCF, this delay was found to be 195 (82,247) days with the highest being 365 days while it was 255 (105, 341) days in private HCF with the highest being 365 days.

One patient who remained in the public sector was diagnosed at his third visit with a delay of 210 days. Similarly, a patient who remained in the private sector was diagnosed after a delay 120 days on his third visit.

Furthermore, median delay among female patients (30 days) was more than male patients (20 days). Also patients aged more than 45 years had more median delay of 30 days as compared to those below 45 years.

The most common reason for shifting from first to second HCF was referral (both public and private) followed by non-affordability (only seen in private HCFs) and non-satisfaction (seen more in private HCFs). Similarly, the reasons for shifting from second to third HCF were referral (mostly in public), non-satisfaction and non-affordability in private. In the third shift, only reason given was referral to DDR-TBC ( Table 1).

**Table 1.** Reasons for shifting from one HCF to other.

Reasons for shifting	Public, n (%)	Private, n (%)
Treatment not available	1 (50)	1 (50)
Treatment not affordable	0 (0)	8 (100)
Referred	13 (54.2)	11 (45.8)
Not satisfied	1 (16.7)	5 (83.3)
Not affordable	0 (0)	2 (100)
Referred	14 (82.4)	3 (17.6)
Not satisfied	1 (33.5)	2 (66.7)

## Discussion

MDR TB is an emerging disease in India. The disease is difficult to treat and treatment outcomes are poor, making it a potential public health threat in the future. Our study sheds light on patients’ treatment pathway and reasons for shifting between health care providers for diagnosis and treatment for MDR TB.

In our study, 63% of patients went to private health care facilities (HCF) as first point of health care contact, which was higher than the average 48% as seen in a systematic review from India ^13, 14^. This pattern is also seen in other studies done in India and in other developing countries ^14– 20^. The health seeking behavior of a patient depends on the knowledge about the disease, severity of symptoms and social support available, especially in a communicable and stigmatized disease like TB ^21^. Studies conducted in India showed that most people had poor awareness about TB-related symptoms, transmission, and the services offered by the then National TB control programme ^22, 23^. Moreover, patients in India have reported treatment barriers, such as, long distance between the TB centers and their homes, lack of confidence in the efficacy of government supplied medication, and the lack of privacy during directly observed treatment sessions ^16, 22, 24^. All these reasons suggest a preference for private HCF as their preferred health provider for TB diagnosis and treatment.

Private sector poses many hurdles in TB control with respect to suboptimal care, lack of standard operating procedures in diagnosis and treatment, lack of accountability as compared to public sector ^25– 27^.

However, there was a delay on the part of patients to report to their first point of health care contact after appearance of symptoms. The median delay was found to be 20 days which is slightly more than two weeks of the cough criterion issued by NTEP for TB screening. While comparing with other studies, the delay varied across the country, some studies found this delay more as compared to others ^16, 26– 31^. This shows the diversity in patient delay across India. Also, the median delay was found to be more among female patients as compared to male patients. This has been shown in various studies done in India as well as in other developing countries ^18, 12, 32^. The median delay was found to be greater among those who had public HCF as their first point of contact which has also been seen in the study done by Nimbarte *et al*. ^27^ This could be due to their procrastinating the health care contact.

In our study, (23/40) 57.5% of the patients were diagnosed at their first point of contact, while (14/17) 82.3% them were diagnosed at their second point of contact. The results are similar to those seen in the study done by Ananthakrishnan *et al*. ^30^ Among the 55% of patients who made a second visit to a different HCF other than DDR-TBC, two thirds of the patients went to a public HCF. This is also seen in the study done by Charles *et al.* ^32^ Also we found that in all the visits, the rate of diagnosis at public HCF was always more than that at private HCF. This could be due to improper tests done for detection or lack of technologies at private HCF ^33^.

The most common reason for shifting between HCF other than referrals was unaffordability followed by dissatisfaction. This is in contrast to the study done by Charles *et al.* where the major reason was found to be dissatisfaction with the available HCF followed by unaffordability ^32^. In public HCF, the most common reason was referral which was in accordance to the PMDT guidelines.

## Conclusion

The present study found that there was substantial patient delay and total delay in diagnosis and treatment of DR TB patients. Private HCF was first point of contact for most of the patients, however the diagnostic rate was high in public HCF. The study projects the need of a public-private collaboration in treating DR TB cases; in terms of linkages between public and private sector for diagnosis and treatment of drug resistance TB, provisions of standard diagnostic services and treatment at low to no costs. This may be achieved by incentivizing treatment and providing standard diagnostic modalities to private sector under NTEP.

**Limitations-** This study was conducted among the 40 patients from one DDR-TBC of Karnataka therefore the findings can be generalized to the population seeking health care from the same DDR-TBC. Second limitation would be the subject variability of the definition, however we have made full effort to validate the findings with medical reports of the patients from DDR-TBC to ensure the starting and end point of the pathway. Since the study was based on recall of the patients, there are chances of inherent recall bias.

## Data availability

### Underlying data

According to the IEC of Medical College Mangalore, we are not permitted to share data with any external agency for protection of data as it contains information which is personal and can be identified. However, if required by anyone, the data can be requested from the corresponding author after full justification of usage of the information. Conditions of access: researchers must use the data for similar research or sufficiently anonymize and give due credit to the authors of this study.

### Extended data

Open Science Framework: Patient treatment pathways of multidrug-resistant tuberculosis cases in coastal South India: Road to a drug resistant tuberculosis centre: Structured questionnaire, https://doi.org/10.17605/OSF.IO/TQNUE ^34^

Data are available under the terms of the Creative Commons Zero “No rights reserved” data waiver (CC0 1.0 Public domain dedication).
