# Supplementary material for: Patient treatment pathways of multidrug-resistant tuberculosis cases in coastal South India: Road to a drug resistant tuberculosis center
Source: F1000Res. 2021 Jul 8;8:498. Originally published 2019 Apr 16. [Version 5] doi: 10.12688/f1000research.17743.5 (PMC8112457; doi:10.12688/f1000research.17743.5)
Supplement: Peer review history

# Peer review: Reviewer response for version 5

**DOI:** 10.5256/f1000research.58432.r89238

**Published:** 2021-07-12

**Authors:** Derrick Kimuli

**Affiliations:** 1 Uganda Bureau of Statistics, Kampala, Uganda

Thank you for responding to my previous comments. Ensuring clarity so that the reader is able to understand the context of the article from the Author's point of view is important. Thanks, again. 

 Just one minor comment on Paragraph 3, " Private sector poses many hurdles in TB control with respect to suboptimal care, lack of standard operating procedures in diagnosis and treatment, lack of accountability as compared to public sector".

 This just should be joined logically to either the previous or the proceeding paragraph.

Is the work clearly and accurately presented and does it cite the current literature?

Yes

If applicable, is the statistical analysis and its interpretation appropriate?

Yes

Are all the source data underlying the results available to ensure full reproducibility?

No

Is the study design appropriate and is the work technically sound?

Partly

Are the conclusions drawn adequately supported by the results?

Partly

Are sufficient details of methods and analysis provided to allow replication by others?

Yes

Reviewer Expertise:

Public Health Research

I confirm that I have read this submission and believe that I have an appropriate level of expertise to confirm that it is of an acceptable scientific standard.

# Peer review: Reviewer response for version 5

**DOI:** 10.5256/f1000research.58432.r89239

**Published:** 2021-07-09

**Authors:** Sharath Burugina Nagaraja

**Affiliations:** 1 Department of Community Medicine, Employees State Insurance Corporation Medical College and Post Graduate Institute of Medical Sciences and Research, Bengaluru, Karnataka, India

No further comments to make.

Is the work clearly and accurately presented and does it cite the current literature?

Yes

If applicable, is the statistical analysis and its interpretation appropriate?

Yes

Are all the source data underlying the results available to ensure full reproducibility?

Yes

Is the study design appropriate and is the work technically sound?

Yes

Are the conclusions drawn adequately supported by the results?

Yes

Are sufficient details of methods and analysis provided to allow replication by others?

Yes

Reviewer Expertise:

Tuberculosis, HIV, Diabetes, COVID

I confirm that I have read this submission and believe that I have an appropriate level of expertise to confirm that it is of an acceptable scientific standard.

# Peer review: Reviewer response for version 4

**DOI:** 10.5256/f1000research.28235.r84550

**Published:** 2021-05-10

**Authors:** Derrick Kimuli

**Affiliations:** 1 Uganda Bureau of Statistics, Kampala, Uganda

**General comment: **

- This is an informative paper. Understanding the points of delay is essential in developing approaches to improve the diagnosis and treatment of DR-TB. However, the paper could use a more in-depth approach to the discussion of the findings. I state some examples below but this could be considered a general comment. 

**Second last paragraph: **

- The poor outcomes notice in India could also be a consequence of the findings of this study. 

**Discussion: **

- Paragraph 2: You may need to elaborate more clearly as to why the first point of health contact was Private HCFs. Could this be attributed to the fact that they have a higher reach than Public HCFs? It is also possible that the Private HCFs are not able to handle MDR TB treatment or could it be that they can but it is very expensive? This doesn't come out well in the discussion yet it is part of the conclusion.

 

**Conclusion: **

- "provisions of standard diagnostic services and treatment at low to no costs" is hardly possible. You could make more precise and realistic recommendations based on your understanding of the context area. 

**Other comments: **

- You could use some additional language editing.

Is the work clearly and accurately presented and does it cite the current literature?

Yes

If applicable, is the statistical analysis and its interpretation appropriate?

Yes

Are all the source data underlying the results available to ensure full reproducibility?

No

Is the study design appropriate and is the work technically sound?

Partly

Are the conclusions drawn adequately supported by the results?

Partly

Are sufficient details of methods and analysis provided to allow replication by others?

Yes

Reviewer Expertise:

Public health, TB

I confirm that I have read this submission and believe that I have an appropriate level of expertise to confirm that it is of an acceptable scientific standard, however I have significant reservations, as outlined above.

# Response

**Published:** 2021-07-05

**Authors:** Priya Rathi

Reviewer’s comment

 Authors reply

 Line/paragraph

 The poor outcomes notice in India could also be a consequence of the findings of this study.

 Dear Reviewer- This paragraph is the justification of conducting this study based on existing evidence prior to the study, hence we have not added the finding of the study in the introduction paragraph.

 Introduction-  Second last paragraph

 Discussion:

 Paragraph 2: You may need to elaborate more clearly as to why the first point of health contact was Private HCFs. Could this be attributed to the fact that they have a higher reach than Public HCFs? It is also possible that the Private HCFs are not able to handle MDR TB treatment or could it be that they can but it is very expensive? This doesn't come out well in the discussion yet it is part of the conclusion.

 Thank you for the suggestions, we have improvised in the justification and discussion on why the first point of contact would be the private HCFs incorporating the points suggested.

 Availability of the healthcare services, ease of access, and less complicated registration process in the private health care sector,

 We also incorporated lack of the expertise and cost required to diagnose and manage MDRTB in the private sector as the reasons for shifting to the public sector on later visits.

 Discussion – Paragraph 2

 Conclusion:

 "provisions of standard diagnostic services and treatment at low to no costs" are hardly possible. You could make more precise and realistic recommendations based on your understanding of the context area

 Dear Reviewers, In India, standard diagnostic services and no-cost treatment have been provided to all MDRTB registered patients.

 But they must bear the cost of management at private HCF, In conclusion, we are recommending a mixed approach wherein the private sector can provide the same treatment funded by the government.

 Conclusion.

# Peer review: Reviewer response for version 3

**DOI:** 10.5256/f1000research.26824.r63971

**Published:** 2020-07-13

**Authors:** Douglas Fraser Wares

**Affiliations:** 1 KNCV Tuberculosis Foundation, The Hague, The Netherlands

**Second review** (12/07/20) of “Patient treatment pathways of multidrug-resistant tuberculosis cases in coastal South India: Road to a drug resistant tuberculosis center” paper for F1000Research

 The authors have answered the vast majority of the comments raised in the earlier versions. However, the are a number of comments which could be addressed if possible prior to full approval. My major and minor comments follow, namely:

**Major comments:**

1. In the flowchart and text, the authors talk about “Presumptive MDR-TB patients”. However, the patients enrolled in the study are diagnosed and registered MDR-TB cases. These are not the same thing. How is “Presumptive MDR-TB patients” defined and is the same definition being used in both the private and public sector facilities in the study?
*The above has been addressed by the authors in the revised version. However the second query of “whether similar levels of identification of “Presumptive MDR-TB patients” was being done in the different sector facilities?”, has not been answered. If it is possible, this could be added.*
2. Time to diagnosis will be determined to a degree by what diagnostic capacity is available in the respective health facility. Hence if a facility has the capacity for rapid molecular diagnosis of TB and rifampicin resistance (RR-TB) e.g. if it has a GeneXpert machine on site, then diagnosis of RR-TB can be done within the day. If not and the patient or sample has to be sent to another facility which then does only culture and phenotypic drug susceptibility testing (DST) and not rapid molecular DST, there will be an inherent time difference between the patients seen at the initial health facility. So did all the health facilities involved in the study have the same access to diagnostic capacity?
*Although the authors mention that the diagnostic capacity is not the same across the health facilities and sectors, no details are included. If it is possible to add details, then this should be done. *
3. Even if the diagnostic capacity available to all health facilities involved in the study, where any RR-/MDR-TB cases diagnosed but not registered? If yes, were the proportions the same in the different sectors analysed?
*Crucial segments of the author’s response to the above query should be added to the actual manuscript.*
4. Were the 15 DR-TB patients who did not consent to participate in the study similar to the 40 patients who did participate in the study? Was there any difference in these 15 patients between those who initially visited a public sector facility versus those who visited a private facility initially?
*Crucial segments of the author’s response to the above query should be added to the actual manuscript. A number of data should be available on these 15 patients (e.g. sex, age, etc) from the records and could be included in the manuscript. *

**Minor comments:**

1. Introduction, 1 ^st^ paragraph, 2 ^nd^ sentence: Is the use of “.. incidence rate”… correct here?
2. Methods, study setting, 1 ^st^ paragraph, 2 ^nd^ sentence: “.. six lakhs..” should be amended to “600,000” for non-Indian readers understanding.
3. Methods, data collection, 3 ^rd^ sentence, reference 10: Is this not the same reference as used for number 4?
4. Results, details of the first visit, and shift from one HCF to another HCF, 2 ^nd^ paragraph, 1 ^st^ sentence: Is it more correct to say that the patients were “referred” to the DDR-TBC rather than “transferred’ as they had not yet started on treatment?  
5. Results, Time delay in treatment pathway of MDR patients: I do not see mention of total delays here?
6. Results, Time delay in treatment pathway of MDR patients, 5 ^th^ paragraph: Better to use “longer” rather than “more”.
7. Results, Table 1: Why are a number of the lines repeated (i.e. Treatment not affordable/Not affordable; Referred; Not satisfied) as there is no explanation to why they appear twice?
8. Discussion, 4 ^th^ paragraph, 2 ^nd^ sentence: Is the median delay not “25 days”?
9. Discussion, 4 ^th^ paragraph, 6 ^th^ sentence: Is it meant to be stated as references “18, 12, 32”.
10. Conclusions, 2 ^nd^ sentence: Not sure that “diagnostic rate” is the correct term to be used here as it has not been calculated in the results section?
11. Limitations, 1 ^st^ sentence: Amend to “ … the findings can *only *be generalized..”

Is the work clearly and accurately presented and does it cite the current literature?

Yes

If applicable, is the statistical analysis and its interpretation appropriate?

Yes

Are all the source data underlying the results available to ensure full reproducibility?

Yes

Is the study design appropriate and is the work technically sound?

Partly

Are the conclusions drawn adequately supported by the results?

Partly

Are sufficient details of methods and analysis provided to allow replication by others?

Yes

Reviewer Expertise:

Drug resistant TB, implementation of new and/or novel drug regimens

I confirm that I have read this submission and believe that I have an appropriate level of expertise to confirm that it is of an acceptable scientific standard, however I have significant reservations, as outlined above.

# Response

**Published:** 2020-07-20

**Authors:** Priya Rathi

**Reviewer’s Name -   Douglas Fraser Wares**

 In the flowchart and text, the authors talk about “Presumptive MDR-TB patients”. However, the patients enrolled in the study are diagnosed and registered MDR-TB cases. These are not the same thing. How is “Presumptive MDR-TB patients” defined and is the same definition being used in both the private and public sector facilities in the study?

 The above has been addressed by the authors in the revised version. However, the second query of “whether similar levels of identification of “Presumptive MDR-TB patients” was being done in the different sector facilities?”, has not been answered. If it is possible, this could be added.

 This study was a retrospective inquiry on the pathway taken to reach Drug Resistance TB Center, which is run by the Government.

 The starting point of this inquiry was taken from the time these patients were presumptive DR case which by definition as per Programmatic Management of Drug Resistant Tuberculosis Guidelines is:

 Presumptive MDR case is any of the following:  and it refers to the following patients in order of their risk: 

 TB patients found positive on any follow-up sputum smear examination during treatment with first line drugs including treatment failures; - TB patients who are contacts of DR-TB; - previously treated TB patients; - new TB patients with HIV co-infection. The point of inquiry started from the time Patients fulfilled any of the above criteria. Since we have not interviewed any of the private HCF we cannot comment if they used the same criteria.

 Method –Data collection, Operational definition.

 Time to diagnosis will be determined to a degree by what diagnostic capacity is available in the respective health facility. Hence if a facility has the capacity for rapid molecular diagnosis of TB and rifampicin resistance (RR-TB) e.g. if it has a GeneXpert machine on site, then diagnosis of RR-TB can be done within the day. If not and the patient or sample has to be sent to another facility which then does only culture and phenotypic drug susceptibility testing (DST) and not rapid molecular DST, there will be an inherent time difference between the patients seen at the initial health facility. So did all the health facilities involved in the study have the same access to diagnostic capacity?

 Although the authors mention that the diagnostic capacity is not the same across the health facilities and sectors, no details are included. If it is possible to add details, then this should be done.

 Not all the health facilities involved in the study have the same diagnostic capacity. We have tried to highlight the same in our study; i.e. those patients who visited such health care facilities, which lacked standard diagnostic facilities were diagnosed late as compared to those who visited the government health care facility which has better diagnostic facility with Standard Operating Procedures (SOPs) and standard of care with respect to DR-TB. Under PMDT programme, DDR- TB centers are developed which complies with Standard Diagnostic and Treatment guidelines. The private practitioners or other small healthcare establishments which are not designated microscopic centers either refer the patient or send the samples to DMCs for diagnosis.

 The relevant text has been added in manuscript in the result section

 Sdjhje

 Even if the diagnostic capacity available to all health facilities involved in the study, where any RR-/MDR-TB cases diagnosed but not registered? If yes, were the proportions the same in the different sectors analysed?

 Crucial segments of the author’s response to the above query should be added to the actual manuscript.

 We have only considered those patients who were registered at DDR- TB Center and enquired which health care facilities they visited prior to DDR-TB Center and their sequence to what happened there with respect to diagnosis and treatment and referral.

 For the patients in the government sector, registration is done under the National TB Program. For patients who are initiated with treatment in the private sector, the private sector notifies this to the government. In this study, our objective was to find out the pathway and the source of information was the patient themselves and not the health care providers. Hence, we did not enquire on the notification of the cases if diagnosed at private sector. . For the patients in the government sector, registration is done under National Tuberculosis Elimination Programme (NTEP). For patients who are initiated with treatment in the private sector, are notified to the government using a the web enabled patient management system for TB control called as Nikshay. Cases which are notified is kept confidential and can be accessed only by appropriate government officials at state and national level

 We have now added the relevant text in main manuscript

 Method –Data collection, Operational definition

 Were the 15 DR-TB patients who did not consent to participate in the study similar to the 40 patients who did participate in the study? Was there any difference in these 15 patients between those who initially visited a public sector facility versus those who visited a private facility initially?

 Crucial segments of the author’s response to the above query should be added to the actual manuscript. A number of data should be available on these 15 patients (e.g. sex, age, etc) from the records and could be included in the manuscript.

 Since the direction of inquiry was retrospective and the unit of inquiry was the patient or close relative, in the 15 patients who did not consent for the study, we were unable to find their first point of contact to Health care facility.

 However, with respect to current diagnosis of TB and treatment regimen, they were same as both were diagnosed at DRTB center with standard protocol and SOP.

 Result paragraph 1

 Introduction, 1st paragraph, 2nd sentence: Is the use of “. incidence rate” … correct here?

 Yes they developed TB in 2017 which was reported in the global report., however we have changed the sentence to avoid confusion

 Introduction para 1

 Methods, study setting, 1st paragraph, 2nd sentence: “... six lakhs.” should be amended to “600,000” for non-Indian readers understanding.

 Done

 Methods, study setting, 1st paragraph, 2nd sentence:

 Methods, data collection, 3rd sentence, reference 10: Is this not the same reference as used for number 4?

 Yes, we Agree and we have now changed it to ref 4

 Methods, data collection, 3rd sentence

 Results, details of the first visit, and shift from one HCF to another HCF, 2nd paragraph, 1st sentence: Is it more correct to say that the patients were “referred” to the DDR-TBC rather than “transferred’ as they had not yet started on treatment? 

 We have changed the word transferred to referred

 Results, details of the first visit, and shift from one HCF to another HCF, 2nd paragraph, 1st sentence

 Results, Time delay in treatment pathway of MDR patients: I do not see mention of total delays here? Added

 We have now added total delays as well the following has been added to the manuscript

 The total median(IQR) delay inclusive of health care delay among all patient was 237 (109, 491) days. The total median (IQR) delay among those who first visited private HCF was 455 (149,505) days, and those who first went to public HCF was 165 (105,410) days.

 Result, time delay --- paragraph 1

 Results, Time delay in treatment pathway of MDR patients, 5th paragraph: Better to use “longer” rather than “more”.

 We have used “longer” instead of “more”.

 Results, Time delay in treatment pathway of MDR patients, 5th paragraph

 Results, Table 1: Why are a number of the lines repeated (i.e. Treatment not affordable/Not affordable; Referred; Not satisfied) as there is no explanation to why they appear twice?

 The sub heading  of the tables were missing, we have now included that.

 Table 1

 Discussion, 4th paragraph, 2nd sentence: Is the median delay not “25 days”

 Yes, we changed it to 25 days now

 Discussion, 4th paragraph, 2nd sentence

 Discussion, 4th paragraph, 6th sentence: Is it meant to be stated as references “18, 12, 32”.

 We have now removed ref 32 for this sentence and have interchanged the sequence in citation

 Conclusions, 2nd sentence: Not sure that “diagnostic rate” is the correct term to be used here as it has not been calculated in the results section?

 We have now removed the word diagnostic rate from conclusion and have added the following statement “those who approached public HCF were diagnosed earlier as compared to others”

 Conclusion, 2 ^nd^ sentence

 Limitations, 1st sentence: Amend to “ … the findings can only be generalized..”

 We have added the word “only”

# Peer review: Reviewer response for version 3

**DOI:** 10.5256/f1000research.26824.r63972

**Published:** 2020-05-29

**Authors:** Sharath Burugina Nagaraja

**Affiliations:** 1 Department of Community Medicine, Employees State Insurance Corporation Medical College and Post Graduate Institute of Medical Sciences and Research, Bengaluru, Karnataka, India

The authors have addressed my earlier comments and I have no further comments.

Is the work clearly and accurately presented and does it cite the current literature?

Yes

If applicable, is the statistical analysis and its interpretation appropriate?

Yes

Are all the source data underlying the results available to ensure full reproducibility?

Yes

Is the study design appropriate and is the work technically sound?

Yes

Are the conclusions drawn adequately supported by the results?

Yes

Are sufficient details of methods and analysis provided to allow replication by others?

Yes

Reviewer Expertise:

Tuberculosis, HIV, Diabetes - Operational Research

I confirm that I have read this submission and believe that I have an appropriate level of expertise to confirm that it is of an acceptable scientific standard.

# Response

**Published:** 2020-05-29

**Authors:** Priya Rathi

We thank Dr Sharath B for helping us in improving the manuscript and final approval of the same.

Dr Priya Rathi with all Co-authors

# Peer review: Reviewer response for version 2

**DOI:** 10.5256/f1000research.26326.r63372

**Published:** 2020-05-20

**Authors:** Sharath Burugina Nagaraja

**Affiliations:** 1 Department of Community Medicine, Employees State Insurance Corporation Medical College and Post Graduate Institute of Medical Sciences and Research, Bengaluru, Karnataka, India

The majority of the comments are addressed. However, please find a few minor comments that need your attention:

1. In Study settings: first paragraph, consider using "catchment area" or any other word rather than "drainage". Please rephrase the statement.
2. In conclusion: I am skeptical about the words "diagnostic yield" - the study was not designed to measure the diagnostic yield. Please rephrase the sentence.

Thank you with regards,

 Sharath BN

Is the work clearly and accurately presented and does it cite the current literature?

Yes

If applicable, is the statistical analysis and its interpretation appropriate?

Yes

Are all the source data underlying the results available to ensure full reproducibility?

Yes

Is the study design appropriate and is the work technically sound?

Yes

Are the conclusions drawn adequately supported by the results?

Yes

Are sufficient details of methods and analysis provided to allow replication by others?

Yes

Reviewer Expertise:

Tuberculosis, HIV, Diabetes - Operational Research

I confirm that I have read this submission and believe that I have an appropriate level of expertise to confirm that it is of an acceptable scientific standard, however I have significant reservations, as outlined above.

# Response

**Published:** 2020-05-21

**Authors:** Priya Rathi

1.In Study settings: first paragraph, consider using "catchment area" or any other word rather than "drainage". Please rephrase the statement.

Authors reply -Word “drainage” has been replaced by “inflow” in study setting first paragraph.

2.In conclusion: I am skeptical about the words "diagnostic yield" - the study was not designed to measure the diagnostic yield. Please rephrase the sentence.

Authors reply -“Diagnostic yield” has been replace by “diagnostic rate” in the conclusion.

# Peer review: Reviewer response for version 1

**DOI:** 10.5256/f1000research.19401.r59044

**Published:** 2020-02-06

**Authors:** Douglas Fraser Wares

**Affiliations:** 1 KNCV Tuberculosis Foundation, The Hague, The Netherlands

Review of “Patient treatment pathways of multidrug-resistant tuberculosis cases in coastal South India: Road to a drug resistant tuberculosis center” paper for F1000Research.

 The authors have written about an important topic related to the treatment pathways of drug-resistant TB patients in India. The manuscript reads well, however I have a number of major comments which need to be addressed to ensure the scientific level of the manuscript is raised. My major and minor comments follow, namely:

**Major comments:**

1. In the flowchart and text, the authors talk about “Presumptive MDR-TB patients”. However the patients enrolled in the study are diagnosed and registered MDR-TB cases. These are not the same thing. How is “Presumptive MDR-TB patients” defined and is the same definition being used in both the private and public sector facilities in the study? This needs to be stated by the authors and how the “Presumptive MDR-TB patients” are being identified in the facilities of the 2 sectors and whether similar levels of identification of “Presumptive MDR-TB patients” was being done in the different sector facilities?
2. Time to diagnosis will be determined to a degree by what diagnostic capacity is available in the respective health facility. Hence if a facility has the capacity for rapid molecular diagnosis of TB and rifampicin resistance (RR-TB) e.g. if it has a GeneXpert machine on site, then diagnosis of RR-TB can be done within the day. If not and the patient or sample has to be sent to another facility which then does only culture and phenotypic drug susceptibility testing (DST) and not rapid molecular DST, there will be an inherent time difference between the patients seen at the initial health facility. So did all the health facilities involved in the study have the same access to diagnostic capacity?
3. Even if the diagnostic capacity available to all health facilities involved in the study, where any RR-/MDR-TB cases diagnosed but not registered? If yes, were the proportions the same in the different sectors analysed?
4. Were the 15 DR-TB patients who did not consent to be participate in the study similar to the 40 patients who did participate in the study? Was there any difference in these 15 patients between those who initially visited a public sector facility versus those who visited a private facility initially?

**Minor comments:**

1. Reference 1 and related text could be updated as the WHO 2019 Global TB Report is available.
2. Introduction, 2 ^nd^ paragraph. It should be stated that PMDT is part of the Revised National TB Control Programme (now renamed as the National TB Elimination Programme) and is not a separate programme as implied by the current text.
3. Introduction, 2 ^nd^ paragraph. Reference 5 should be updated as it refers currently to a 2011 WHO document which has been updated since then.
4. Introduction, 3 ^rd^ paragraph. Reference 6 is from a paper dealing with Argentina. Is there no relevant reference paper from India that could be used instead? Also does the current reference paper talk about DS-TB and/or DR-TB?
5. Methods, Study settings, 1 ^st^ paragraph. The district name and hospital name where the PMDT centre is located should be given. As should the names of the 3 districts that the centre caters for.
6. Methods, Study settings, 1 ^st^ paragraph. For non-Indian readers, the meaning of “… by Kayakalp.” needs to be explained. Also the reference given is no. 6 which refers to a paper dealing with Argentina and hence seems incorrect.    
7. Methods, Study settings, 1 ^st^ paragraph. The authors mention “.. DOTS-Plus centres” and only mention about initial in-patient care. DOTS-Plus is outdated terminology and initial in-patient care practice alone is outdated practice under RNTCP. The text should be amended so the reader understand what is current RNTCP terminology and practice. Especially as in Figure 1, the term of DR-TB Centre is used.
8. Methods, Study settings, 2 ^nd^ paragraph. 12.5% of patients in the study were illiterate, hence how was “written consent” given?
9. Methods, Data collection, 1 ^st^ paragraph. Is it correct then that there was no actual field testing of the questionnaire prior to the study being conducted? What training did the PI have on the questionnaire prior to conducting the patient interviews?
10. Methods, Operational definitions, reasons for shifting from one HCF to another, 1 ^st^ paragraph. As pointed out by the previous reviewer, what is listed here are options or answers expected to be provided by the patients. Hence this part of the methods section needs revising.
11. Results, 1 ^st^ paragraph. Need to be consistent in the use of both numbers and % in the last sentence.
12. Results, Details of first visit and shift from one HCF to other HCF, 2 ^nd^ paragraph. Although 12 of the 15 who initially visited a public HCF were diagnosed with MDR, only 10 were immediately referred to the DR-TB centre. Why did the 2 other patients have to attend a HCF for a 2 ^nd^ time before referral to the DR-TB Centre?
13. Discussion. As stated by the previous reviewer, the authors spend a lot of the discussion section comparing with other studies. Many of the times this is just in relation to confirming previous observations. Such text could be reduced (e.g. 2 ^nd^ and 4 ^th^ paragraphs).
14. Discussion, 2 ^nd^ paragraph. Should ease of access to service be more prominent amongst important issues surrounding health seeing behavior of patients? Is this not a major reason why patients initially seek care in the private sector rather than the public sector?
15. Discussion, 3 ^rd^ paragraph. DR-TB is now more of a “transmitted” disease rather than the “acquired resistance” as discussed in the first few sentences of the paragraph. This needs to be noted. Also the meaning of the sentence “Further, the private sector poses many hurdles in TB control.” is unclear?
16. Discussion, 5 ^th^ paragraph. For clarity of reading, the authors should to include the actual numbers involved when they state “57.5%” (=23/40) and “82.3” (=14/17).
17. Discussion, 6 ^th^ paragraph. Did the study by Charles *et al.* (ref 28) include DR-TB cases as well as DS-TB? If not, how relevant is the reference to the current study?
18. Limitations. The authors need to include a paragraph or two on “Limitations” of the study.
19. Conclusions. Some of the statements made are pretty sweeping with little data or evidence from the study to support them. For example the statements in the 1 ^st^ paragraph made about female and the elderly – these are based on very small patients numbers and hence how valid and generalizable are they? In the 2 ^nd^ paragraph, an alternative view is that there may be better awareness in the community of the disease and availability of services. Hence a higher proportion of those who actually have the disease present to the public sector facilities for their 1 ^st^ visit. And surely better linkages between the HCFs of the two sectors needs to be established, in addition to the option of providing everything in all private HCFs?
20. Consistency of language. DOTS-Plus centers / DR-TB Centre / PMDT center are all used in the manuscript. Firstly I would suggest the authors use “centre” and “programme”, and also are consistent in the term they use for the centre where the DR-TB patients were registered.

Is the work clearly and accurately presented and does it cite the current literature?

Yes

If applicable, is the statistical analysis and its interpretation appropriate?

Yes

Are all the source data underlying the results available to ensure full reproducibility?

Yes

Is the study design appropriate and is the work technically sound?

Partly

Are the conclusions drawn adequately supported by the results?

Partly

Are sufficient details of methods and analysis provided to allow replication by others?

Yes

Reviewer Expertise:

Programmatic care and control of tuberculosis; drug-resistant tuberculosis

I confirm that I have read this submission and believe that I have an appropriate level of expertise to confirm that it is of an acceptable scientific standard, however I have significant reservations, as outlined above.

# Response

**Published:** 2020-05-02

**Authors:** Priya Rathi

We have answered all query/comments point to point and have indicated its current location in modified manuscript.

*In the flowchart and text, the authors talk about “Presumptive MDR-TB patients”. However, the patients enrolled in the study are diagnosed and registered MDR-TB cases. These are not the same thing. How is “Presumptive MDR-TB patients” defined and is the same definition being used in both the private and public sector facilities in the study? This needs to be stated by the authors and how the “Presumptive MDR-TB patients” are being identified in the facilities of the 2 sectors and whether similar levels of identification of “Presumptive MDR-TB patients” was being done in the different sector facilities?*

This study was a retrospective inquiry on pathway taken to reach Drug Resistance TB Center. The starting point of this inquiry was taken from the time these patients were presumptive DR case which by definition as per Programmatic Management of Drug Resistant Tuberculosis Guidelines is: Presumptive MDR case are Any of the following:

It refers to the following patients in order of their risk: TB patients found positive on any follow-up sputum smear examination during treatment with first-line drugs including treatment failures; - TB patients who are contacts of DR-TB; - previously treated TB patients; - new TB patients with HIV co-infection.

These definitions were used in the study.

Method –Data collection, Operational definition.

Time to diagnosis will be determined to a degree by what diagnostic capacity is available in the respective health facility. Hence if a facility has the capacity for rapid molecular diagnosis of TB and rifampicin resistance (RR-TB) e.g. if it has a GeneXpert machine on site, then diagnosis of RR-TB can be done within the day. If not and the patient or sample has to be sent to another facility which then does only culture and phenotypic drug susceptibility testing (DST) and not rapid molecular DST, there will be an inherent time difference between the patients seen at the initial health facility. So did all the health facilities involved in the study have the same access to diagnostic capacity?

Not all the health facilities involved in the study have the same diagnostic capacity. This is what we are trying to bring out from our study, that those patients who visited such health care facility which lacked Standard Diagnostic facilities were diagnosed late as compare to those who visited Government Health facility which has better diagnostic facility with Standard Operating Procedures (SOPs) and standard of care with respect to DR-TB. Under PMDT programme, DDR- TB centers are developed which complies with Standard Diagnostic and Treatment guidelines.

*Even if the diagnostic capacity available to all health facilities involved in the study, where any RR-/MDR-TB cases diagnosed but not registered? If yes, were the proportions the same in the different sectors analyzed?*

We have only considered those patients who were registered at DDR- TB Center and enquired which health care facilities they visited prior to DRTB Center and their sequence to what happened there with respect to diagnosis and treatment and referral.

For the patients in the Government Sector, registration is done under the National TB Program. For patients who are initiated with treatment in the private sector, the private sector notifies to the government. In this study, our objective was to find out the pathway and the source of information was the patient themselves and not the health care providers. Hence, we did not enquire on the notification of the cases if diagnosed at private sector.

*Were the 15 DR-TB patients who did not consent to participate in the study similar to the 40 patients who did participate in the study? Was there any difference in these 15 patients between those who initially visited a public sector facility versus those who visited a private facility initially?*

Since the direction of inquiry was retrospective and the unit of inquiry was the patient or close relative, in the 15 patients who did not consent for the study, we were unable to find their first point of contact to Health care facility. However, with respect to the current diagnosis of TB and treatment regimen, they were the same as both were done at DRTB center with standard protocol and SOP.

*Reference 1 and related text could be updated as the WHO 2019 Global TB Report is available.*

We have changed the reference to the latest report.

Reference 1

*Introduction, 2 paragraph. It should be stated that PMDT is part of the Revised National TB*

*Control Programme (now renamed as the National TB Elimination Programme) and is not a*

*separate programme as implied by the current text.*

We have mentioned it now and have highlighted it as well.

Introduction, 2 ^nd^  paragraph.

*Introduction, 2 paragraph. Reference 5 should be updated as it refers currently to a 2011 WHO document which has been updated since then.*

We have updated the same.

Introduction, 2 ^nd^  paragraph

Reference 5.

*Introduction, 3 paragraph. Reference 6 is from a paper dealing with Argentina. Is there no*

*relevant reference paper from India that could be used instead? Also, does the current reference paper talk about DS-TB and/or DR-TB?*

We have not taken any data from the Argentina study, we have just cited the conceptual knowledge of delay in diagnosis, hence the same reference can be used, the study included DS and Previously treated TB patients. We have also added one Indian study as well, however it covers only a few points as compared to Argentina study

Introduction, 3 ^rd^  paragraph. Reference 6 and 7

*Methods, Study settings, 1 paragraph. The district name and hospital name where the PMDT*

*Centre is located should be given. As should the names of the 3 districts that the Centre caters for.*

The DRTB center is situated in district hospital of Dakshina Kannada District, Karnataka State , India which also caters to two neighboring districts of Chikmagalur and Shimoga. This has been included now in the first paragraph.

Methods, Study settings, paragraph 2

*Methods, Study settings, 1 paragraph. For non-Indian readers, the meaning of “… by Kayakalp.”*

*needs to be explained. Also, the reference given is no. 6 which refers to a paper dealing with Argentina and hence seems incorrect.*

This has been removed and the above statement has been mentioned instead.

Methods, Study settings, 2 ^nd^ paragraph

*Methods, Study settings, 1  paragraph. The authors mention “.. DOTS-Plus centers” and only mention about initial in-patient care. DOTS-Plus is outdated terminology and initial in-patient care practice alone is outdated practice under RNTCP. The text should be amended so the reader understands what is current RNTCP terminology and practice. Especially as in Figure 1, the term of DR-TB Centre is used.*

We have changed the terms to District Drug resistance TB center (DDR-TBC) and Peripheral Health Institution (PHI) as per PMDT guidelines.

Methods, Study settings, paragraph 3

*Study settings, 2nd paragraph. 12.5% of patients in the study were illiterate, hence how was “written consent” given*

Those who were illiterate, the informed consent process was conducted in front of literate impartial witness as per ICMR ethics guidelines.( https://www.icmr.nic.in/sites/default/files

/guidelines/ICMR_Ethical_Guidelines_2017.pdf)                                       

Reference 8

*Methods, Data collection, 1st paragraph. Is it correct then that there was no actual field testing of the questionnaire prior to the study being conducted? What training did the PI have on the questionnaire prior to conducting the patient interviews?*

The tool was pilot tested in two patients. PI holds an MD degree in Community Medicine, where there is training in epidemiology and has conducted multiple projects in the past. The questionnaire was content to validate independently by two experts and discussed for feasibility along with the pilot testing.

Not applicable

*Methods, Operational definitions, reasons for shifting from one HCF to another, 1st paragraph. As pointed out by the previous reviewer, what is listed here are options or answers expected to be provided by the patients. Hence this part of the methods section needs revising.*

We have modified the section as Follows-These were the options provided to the patients-1. treatment not available, the appropriate treatment for the symptoms was unavailable in that HCF; 2. treatment not affordable, cost of treatment was beyond the paying capacity of the patient and its family; 3. referred, the patient was asked to visit another HCF for review/consultation or appropriate treatment; 4.not satisfied, symptoms did not alleviate or the patient perceived that the services being provided were inadequate. Also, along with this, they were also asked if any other reasons not included in the list.

Methods- Operational definition

*Result-1st paragraph. Need to be consistent in the use of both numbers and % in the last sentence.*

We have improvised the result with consistency.

Result-1 ^st^  paragraph

*Results, Details of first visit and shift from one HCF to other HCF, 2nd paragraph. Although 12 of the 15 who initially visited a public HCF were diagnosed with MDR, only 10 were immediately referred to the DR-TB center. Why did the 2 other patients have to attend a HCF for a 2nd time before referral to the DR-TB Centre?*

These two patients were from the other districts, they were referred from PHC to District hospital of the concerned district and from there to the DDR-TBC.

*Discussion. As stated by the previous reviewer, the authors spend a lot of the discussion section comparing with other studies. Many of the times this is just in relation to confirming previous observations. Such text could be reduced (e.g. 2nd and 4th paragraphs).*

We have modified the discussion as suggested, we have removed the comparing figures and have only mentioned relevant texts, we have reduced 2 ^nd^ and 4 ^th^ paragraph

Discussion

*Discussion, 2nd paragraph. Should ease of access to service be more prominent amongst important issues surrounding health seeing behavior of patients? Is this not a major reason why patients initially seek care in the private sector rather than the public sector?*

Yes, we have incorporated the point in the discussion

Discussion Paragraph 2

*Discussion, 3rd paragraph. DR-TB is now more of a “transmitted” disease rather than the “acquired resistance” as discussed in the first few sentences of the paragraph. This needs to be noted. Also the meaning of the sentence “Further, the private sector poses many hurdles in TB control.” is unclear?*

There are both kind of cases, acquired as well as transmitted, we have now included your point in our discussion, also we have explained how the private sector can hinder the outcome of DRTB

Discussion paragraph 2

*Discussion, 5th paragraph. For clarity of reading, the authors should to include the actual numbers involved when they state “57.5%” (=23/40) and “82.3” (=14/17).*

Changes have been incorporated

Discussion paragraph 4

*Discussion, 6th paragraph. Did the study by Charles et al. (ref 28) include DR-TB cases as well as DS-TB? If not, how relevant is the reference to the current study?*

We have removed this part of the discussion.

Not applicable

*Limitations. The authors need to include a paragraph or two on “Limitations” of the study.*

Thank you for the insight. We have incorporated the paragraph for limitation.

Limitation reads as:

This study was conducted among the 40 patients from one DDR-TBC of Karnataka therefore the findings can be generalized to the population seeking health care from the same DDR-TBC. Second limitation would be the subject variability of the definition, however we have made full effort to validate the findings with medical reports of the patients from DDR-TBC to ensure the starting and end point of the pathway. Since the study was based on recall of the patients, there are chances of inherent recall bias.

Limitation

*Conclusions. Some of the statements made are pretty sweeping with little data or evidence from the study to support them. For example, the statements in the 1st paragraph made about female and the elderly – these are based on very small patients numbers and hence how valid and generalizable are they? In the 2nd paragraph, an alternative view is that there may be better awareness in the community of the disease and availability of services. Hence a higher proportion of those who actually have the disease present to the public sector facilities for their 1st visit. And surely better linkages between the HCFs of the two sectors needs to be established, in addition to the option of providing everything in all private HCFs*?

We have modified the conclusion based on your suggestion.

Conclusion read as-

The present study found that there was substantial patient delay and total delay in diagnosis and treatment of DR TB patients. The study projects the need of a public-private collaboration in treating DR TB cases; in terms of linkages between public and private sector for diagnosis and treatment of drug resistance TB, provisions of standard diagnostic services and treatment at low to no costs. This may be achieved by incentivizing treatment and providing standard diagnostic modalities to private sector under NTEP.

Conclusion

*Consistency of language. DOTS-Plus centers / DR-TB Centre / PMDT center are all used in the manuscript. Firstly I would suggest the authors use “centre” and “programme”, and also are consistent in the term they use for the centre where the DR-TB patients were registered.*

We have changed it to District drug resistance TB center (DDR-TBC), We have also clarified and changed center and programme.

All throughout the manuscript

# Peer review: Reviewer response for version 1

**DOI:** 10.5256/f1000research.19401.r52457

**Published:** 2019-09-16

**Authors:** Sharath Burugina Nagaraja

**Affiliations:** 1 Department of Community Medicine, Employees State Insurance Corporation Medical College and Post Graduate Institute of Medical Sciences and Research, Bengaluru, Karnataka, India

I congratulate your efforts on working on treatment pathways of drug resistant TB patients. Overall, the manuscript reads well. However, I have few comments for your consideration which will help in betterment of the manuscript. The comments are mentioned separately below.

   

 Comments:

1. The authors in the introduction state that “we aimed to determine the health- seeking pathway of presumptive MDR TB patients prior to treatment at the PMDT center”. The manuscript lacks clarity on identifying the presumptive MDR-TB patient under the programmatic settings. Probably, the authors should specifically say that the presumptive MDR-TB is based on laboratory diagnosis rather than a clinical presumption.
2. Study settings: The authors have to specify the name of the hospital, location of the PMDT centre with the names and population of the districts it is catering to. They should also briefly describe about the types/numbers of public and private health facilities in the districts. 
3. The statement “the patient is released to outpatient care for continuation of care at Directly Observed Treatment (DOT-Plus) centers throughout the community” needs to be modified because under programmatic conditions the patients are referred for domiciliary care in the community. 
4. The name of the institution which has accrued ethics approval needs to be mentioned.
5. Data collection: It is found that the details were collected from the time the patients experience TB symptoms. These details are not analysed and was beyond the scope of the study. For a patient, there is a thin line of difference from being a presumptive TB case and a presumptive DR-TB case. There could be a lot of subjective variability in remembering the things which have occurred in the past. It is better if the authors explain the measures taken to negate such variability during the process of interview and increased their accuracy of findings.
6. Data analysis: Please mention the reference for adobe illustrator trail version.
7. Time delays in health care pathways: I feel the definitions needs further clarity. The starting point for delay calculations is the point of onset of illness. We presume that the authors are taking into account that it is from the point when the patient has a laboratory diagnosis of DR-TB. The authors should explicitly mention in their definitions.
8. Reasons for shifting from one HCF to others: These are the options or answers expected from the patients. They have to be presented in the results as the findings of patients’ interviews. Hence, should be removed from the method section.
9. Results: For better understanding the authors have to describe their findings as numbers (percentages). The ‘p’ values mentioned in table 1 should be removed, it has no relevance.
10. Among the 25 patients who went to private sector, 11 were diagnosed as MDR TB. What type of diagnosis did the private sector facilities made? Clinical or laboratory diagnosis? It is also mentioned that the patients are transferred to PMDT centres. Under programmatic settings, the transfer happens from one RNTCP administrative unit to the other. 
11. There is a scope for authors to present the findings of proportions of patients who underwent 1,2,3 and 4 HCF visits as a bar diagram/table stratified by public and private facilities.
12. Discussions: The authors should remain focused on the study context. There has been much comparison with other studies. This tends to lose the focus of the readers. The recommendations have to be relevant to the study findings.
13. Limitations: The authors can add a paragraph on limitations of the study (response rate, sample size, generalizability, definitions-subjective variability).
14. Conclusion: The conclusion should be specific and should be described in a paragraph.

Is the work clearly and accurately presented and does it cite the current literature?

Yes

If applicable, is the statistical analysis and its interpretation appropriate?

Yes

Are all the source data underlying the results available to ensure full reproducibility?

Yes

Is the study design appropriate and is the work technically sound?

Yes

Are the conclusions drawn adequately supported by the results?

Yes

Are sufficient details of methods and analysis provided to allow replication by others?

Yes

Reviewer Expertise:

Tuberculosis, HIV, Diabetes - Operational Research

I confirm that I have read this submission and believe that I have an appropriate level of expertise to confirm that it is of an acceptable scientific standard, however I have significant reservations, as outlined above.

# Response

**Published:** 2020-05-02

**Authors:** Priya Rathi

*I congratulate your efforts on working on treatment pathways of drug resistant TB patients. Overall, the manuscript reads well. However, I have few comments for your consideration which will help in the betterment of the manuscript. The comments are mentioned separately below.*

We thank the reviewer for the kind words of appreciation and recognising the work behind the manuscript. We have answered all query/comments point to point and have indicated its current location in the modified manuscript.

*The authors in the introduction state that “we aimed to determine the health- seeking pathway of presumptive MDR TB patients prior to treatment at the PMDT centre”. The manuscript lacks clarity on identifying the presumptive MDR-TB patient under the programmatic settings. Probably, the authors should specifically say that the presumptive MDR-TB is based on laboratory diagnosis rather than a clinical presumption.*

We have now included the exact definition of a presumptive diagnosis of DRTB based on PMDT guidelines

Methods data collection  paragraph 2 and operational definition

*Study settings: The authors have to specify the name of the hospital, location of the PMDT centre with the names and population of the districts it is catering to. They should also briefly describe about the types/numbers of public and private health facilities in the districts.*

We have now specified the facility name, location and other details as suggested by the reviewer

Methods. Study setting paragraph 1

*The statement “the patient is released to outpatient care for continuation of care at Directly Observed Treatment (DOT-Plus) centers throughout the community” needs to be modified because under programmatic conditions the patients are referred for domiciliary care in the community.*

We have now changed this based on the PMDT guidelines

Methods. Study setting paragraph 2

*The name of the institution which has accrued ethics approval needs to be mentioned.*

Name of the IEC has been added

Methods. Study setting paragraph 3

*Data collection: It is found that the details were collected from the time the patients experience TB symptoms. These details are not analysed and was beyond the scope of the study. For a patient, there is a thin line of difference from being a presumptive TB case and a presumptive DR-TB case. There could be a lot of subjective variability in remembering the things which have occurred in the past. It is better if the authors explain the measures taken to negate such variability during the process of interview and increased their accuracy of findings.*

We have mentioned the measures taken for alleviating subjectivity as follows

We validated the starting point with the reports and patient file from the DDR-TBC to look for HIV co infection, previous lab reports, history of contact, previous treatment history, if available. 

Data collection

*Data analysis: Please mention the reference for adobe illustrator trail version.*

We have incorporated the reference and link

Data analysis

*Time delays in health care pathways: I feel the definitions needs further clarity. The starting point for delay calculations is the point of onset of illness. We presume that the authors are taking into account that it is from the point when the patient has a laboratory diagnosis of DR-TB. The authors should explicitly mention in their definitions.*

We have changed the definition to make it clearer to understand.

Operational Definitions

*Reasons for shifting from one HCF to others: These are the options or answers expected from the patients. They have to be presented in the results as the findings of patients’ interviews. Hence, should be removed from the method section.*

These were the options given to the patients hence we have mentioned in both the sections

Results:

*For better understanding, the authors have to describe their findings as numbers (percentages). The ‘p’ values mentioned in table 1 should be removed, it has no relevance.*

We have included the numbers and also removed p-value from table 1

*Among the 25 patients who went to private sector, 11 were diagnosed as MDR TB. What type of diagnosis did the private sector facilities made? Clinical or laboratory diagnosis? It is also mentioned that the patients are transferred to PMDT centres. Under programmatic settings, the transfer happens from one RNTCP administrative unit to the other.*

There are private sectors with diagnostic facilities, hence the diagnosis refers to laboratory diagnosis. We meant, that the patient was referred to the public sector from the private sector

***Details of first visit and shift from one HCF to other HCF*** 

Paragraph 3

*There is a scope for authors to present the findings of proportions of patients who underwent 1,2,3 and 4 HCF visits as a bar diagram/table stratified by public and private facilities.*

Since our objective was to map the pathway, we have incorporated the different color lines for different number of visit (1,2,3,4)

This has been explained in the data analysis section as follows

The meanings of both the lines as well the colours have been explained in the legends accompanying the pathways. Moreover, the number of patients shifting between HCF has been represented using the numbers accompanying the respective lines. 

Data-analysis

Figure 2 legends

*Discussions: The authors should remain focused on the study context. There has been much comparison with other studies. This tends to lose the focus of the readers. The recommendations have to be relevant to the study findings.*

We have now removed the comparing figures and have kept only relevant text

Discussion

*Limitations: The authors can add a paragraph on limitations of the study (response rate, sample size, generalisability, definitions-subjective variability).*

Thank you for your insight. We have incorporated the paragraph for limitation.

Limitation reads as

This study was conducted among the 40 patients from one DDR-TBC of Karnataka therefore the findings can be generalized to the population seeking health care from the same DDR-TBC. Second limitation would be the subject variability of the definition, however we have made full effort to validate the findings with medical reports of the patients from DDR-TBC to ensure the starting and end point of the pathway. Since the study was based on recall of the patients, there are chances of inherent recall bias.

Limitation

*Conclusion: The conclusion should be specific and should be described in a paragraph.*

We have made it specific and conveyed it in a single paragraph.

Conclusion read as-

The present study found that there was substantial patient delay and total delay in diagnosis and treatment of DR TB patients. The study projects the need of a public-private collaboration in treating DR TB cases; in terms of linkages between public and private sector for diagnosis and treatment of drug resistance TB, provisions of standard diagnostic services and treatment at low to no costs.This may be achieved by incentivising treatment and providing standard diagnostic modalities to private sector under NTEP.
